# Dr. Gardner and New York Academy of Medicine–Consultations with Homœopathists

**Published:** 1867-12

**Authors:** 


### Editorial Department.

### Dr. Gardner and New York Academy of Medicine--Consultations with Homœopathists.

Dr. Gardner has recently been suspended from membership in the New York Academy, charged with repeated consultations with a homœopath. Dr. Gardner makes no denial of the offense; says, under like circumstances, he would repeat it. The Academy, could, it seems to us, have consistently done nothing less, and Dr. Gardner, though a prominent and highly respectable physician, cannot afford to bear the odium which is certain to follow the course he has pursued. It is inconsistent and absurd on both sides, and very difficult to see why either physician or homeopath should consent to holding a consultation with each other. The *Tribune*, *Times*, &c., &c., have undertaken the defense of Dr. Gardner, but their articles all show profound ignorance of the real question, and go but a very little ways towards justification. We are highly gratified in having all who wish it, able to obtain advice and treatment from a homœopathist—should feel very sorry if any should be deprived of such gratification; but our sympathies would be much greater for the homeopathist if asked to mix regular medicine with his "thirtieth dilution." It would, undoubtedly, entirely subvert the effect of the dilution, while certainly the regular medicine would operate as usual. It would be the unkindest and meanest thing conceivable to thus interfere in the operations of a system of [illegible]

that most cases of disease which terminate favorably under the best treatment, would sooner or later thus terminate without treatment, that care, as well as cure of the sick, is the legitimate sphere of medical practice.

Our intelligent and venerable medical friend has mixed up with his Orthopathic doctrine, and sanctioned, the swindle of bread pills, &c., &c. He is a very religious and honest man with well people, but when his friends grow sick, he fools and imposes upon them bread pills and other inert compounds, showing his honesty by subsequently acquainting them with the cheat—the exposure being greatly to his credit. Physicians do this frequently, and continue the deception, but it is down right quackery and imposition; is a disgrace to the regular profession, and has lowered it to a level with similar impositions.

If there is any "upward upright tendency" in human nature at all, it will soon manifest itself by contempt for the low tricks of unwashed villany, and respect for honest, candid, truthful representations of the nature of disease and its means of cure. Physicians have given bread pills and colored water, homœopathists sugar pills and sugar water; and other quacks have imitated both, and combined their impositions until common honesty requires a change.

We hope no one will wait very long after this, to know what the Buffalo Medical and Surgical Journal has to say upon the downright, downward, damnable tendency of dishonesty, practiced upon people who are sick. Dr. Jennings will please accept our warmest personal thanks for both letter and book. We cannot publish the letter for want of space.

Transactions of the American Medical Association, Vol. xviii, 1867.

The minutes of the Association at its last meeting have been published in the *Journal*, and require no notice. The first paper, then, in the volume not already before our readers, is the Address of the President, Dr. Henry F. Askew, which is well chosen and appropriate. His suggestions are valuable, essential we might say, to the prosperity of the Association and profession. He manifests the highest sense of professional honor, and urges upon the profession the "avoidance of all narrow selfishness, and boastful pretension," and makes suggestions of so vital importance upon many subjects, that we hope every physician will carefully read the Address. The volume also contains the following reports and papers:

Report of the Section on the Practice of Medicine and Obstetrics.

Extra-Uterine Fœtation and Gestation, and the Early Signs which characterize it. By Stephen Rogers, M. D., New York.

Remarks on Heart Diseases as observed in the Military Service from 1861 to 1865 inclusive. By M. K. Taylor, M. D.

Report on the Section of Meteorology, Medical Topography, and Epidemic Diseases.

Report on Meteorology, Medical Topography, and Epidemic Diseases of Illinois. By R. C. Hamill, M. D., Chicago, Ill.

Report of the Section on Surgery.

On the Action of Belladonna in Disease of the Cornea. By Joseph S. Hildreth, M. D., of Chicago, Ill.

A Report on the Use of Plaster of Paris in Surgery. By James L. Little, M. D.

Ligation with Depletion of Varicose Veins of the Leg, with a Case of Radical Cure. By B. Howard, M. D., New York.

Report of the Committee on Ligature of the Subclavian Artery. By Willard Parker, M. D., New York.

A Contribution to the History of the Hip-Joint Operations performed during the late Civil War: being the Statistics of Twenty Cases of Amputations and Thirteen Resections at this Articulation in the Southern Service. By Paul F. Eve, of Nashville, Tenn.

A Statistical Table on Lumbar Colotomy (Amussat's Operation) for the Relief of Non-Congenital Obstruction and of Vesico-Intestinal Fistula. By George C. Blackman, M. D.

[illegible]

Report of the Committee on Medical Literature. By Alfred C. Post, M. D., New York.

Report on Insanity. By Isaac Ray, M. D., late of Providence, R. I.

Report of the Special Committee to whom were referred the several Reports and Papers presented to the Meeting of the American Medical Association during its Session in Cincinnati, May, 1867, and not acted upon by the appropriate Sections, By N. S. Davis, M. D.

Report of the Committee on Local Anæsthesia. By Ernest Krackowizer, M. D., of New York.

Epidemic Cholera: its Causes and the Means for its Prevention. By Elisha Harris, M. D., of New York.

Prize Essay.—On the Cause of Intermittent and Remittent Fevers. By J. R. Black, M. D., of Newark, Ohio.

Prize Essay.—On the Treatment of Certain Uterine Abnormities. By Montrose A. Pallen, M. D., of St. Louis, Mo.

Plan of Organization for a National Medical Association.

Code of Ethics of the American Medical Association.

The volume, as a whole, is unusually creditable to the Association. The papers and reports are highly interesting, and will be found instructive to every member of the profession.

The Secretaries of the Association and the Publishing Committee, have discharged the duties of their offices with great fidelity. The Association and profession certainly owe them a vote of hearty thanks.

---

The Physician's Hand-Book for 1868. By Wm. Elmer M. D., New York: W. A. Townsend and Adams.

This pocket record, which now makes its eleventh annual appearance, has been received with marked favor by the profession; and the several improvements and corrections to which the present edition has been subjected, will certainly win for it increased patronage. As it appears now, we have no doubt that it will fullfil its intended mission of facilitating the daily work of the physician, in the highest degree. The Record of Practice will especially commend itself, having been so arranged that all the names occurring in a physician's practice can be alphabetically recorded.

The Physiology of Man; designed to represent the existing state of Physiological Science as applied to the functions of the Human Body. By Austin Flint, jr., M. D., Professor of Physiology and Microscopy in the Bellevue Hospital Medical College, New York, and in the Long Island College Hospital, etc., etc. Alimination, Digestion, Absorption, Lymph and Chyle. New York: D. Appleton & Co., 1867.

A little over a year ago we had occasion to notice the first volume of this series, in the preface of which the author announced his intention of issuing the work in four separate parts, according to the natural sub-division of the science of physiology, hoping by this plan to be enabled to accomplish his task more efficiently and with a greater degree of elaborateness than could otherwise be attained. The great ability with which the discussions in the first volume have been carried out has prepared the profession for a just appreciation of the succeeding volumes, the second of which we have now before us.

The subjects presented in the present volume, are the functions of regeneration of the animal organism, is a division of physiology certainly of the greatest impertance and interest, and deserving of the careful investigation which it has received at the hands of Dr. Flint. With few exceptions, physiologists generally have not given that prominence which the processes converting matter into organized structures deserves, and a minute acquaintance with which is acknowledged by the profession to be vital in the successful practice of medicine.

The chapters devoted to the consideration of alimenation are very complete and systematic. The remarks made upon the phenomena of hunger and thirst and the description of the proximate principles will not fail to interest and instruct the student, many valuable ideas and practical suggestions being made throughout the text. The effect of improper and insufficient alimentation has received a careful investigation, the author being enabled to incorporate in the same the observations made by Prof. Jos. Jones in charge of the Andersonville Prison, in which some important physiological facts are presented. Contrary to the commonly received opinions that typhus fever is epidemic, where men are subjected to crowding and filth, as well as defective nutrition, this disease was entirely unknown, while contagious fevers were of rare occurrence. Dysentery, diarrhœa, scurvy and hospital gangrene were the prevalent diseases, and the main causes of the extraordinary mortality of hospital gangrene demanded the most serious and earnest investigation, a large number of operations requiring to be performed on account of slight injuries and abrasures which were followed by gangrene, a large proportion of these terminating fatally. In almost all post-mortem examinations Dr. Jones found, more or less, serious effusion into the abdominal cavity. The views entertained by the author, regarding alcoholic stimulants, are in accordance with the present received opinions, namely: that they cannot, physiologically considered, be regarded as alimentary principles, but that their principal effects upon the organism is an exaltation of the nervous system; yet he does not admit that it increases the capacity for endurance of severe and protracted bodily excertions, summing up its influence as a therapeutical agent, "in promoting assimilation in certain conditions of defective neutrition, in relieving shock and nervous exhaustion, in sustaining the powers of life, in acute diseases, characterized by rapid emaciation and abnormally active and destructive assimilation." Succeeding the general remarks upon alcohol, we find analytical investigations of the various distilled and fermented liquors, as also an investigation of the physiological effects of coffee, tea and chocolate, which will be found of practical and scientific value.

The mechanical process of digestion has received a most satisfactory and complete description. The process of emesis is shown to depend mainly upon the diaphragmatic compression of the stomach. The various processes of digestion are considered under the heads of Salivary, Gastric, Pancreatic, Biliary and Intestinal.

There are many other subjects discussed in this volume which we have not space to notice, the completeness of the work leaving nothing within its scope which does not receive exhaustive investigation.

---

The Base of the Brain, with nerves emerging. By S. W. Wetmore, M. D., Demonstrator of Anatomy in the University of Buffalo, N. Y.

This chart is a representation of the cranial nerves, illustrated by a wood cut of the base of the brain. The descriptions of the origin, foramen of exit from skull, principal distribution and functions of the nerves are arranged in a tabular form, presenting this intricate portion of anatomy in the most convenient manner for study. We could wish to see this chart in the possession of every medical student, upon whom Dr. Wetmore has certainly conferred a great favor in presenting this subject in such a clear, systematic and concise manner.

---

Biennial Retrospect of Medicine and Surgery and the Allied Sciences. Edited by Mr. H. Power, Dr. Anstie, Mr. Holmes, Mr. Thomas Windsor, Dr. Barnes, and Dr. C. Hilton Fagge, for the New Sydenham Society. Philadelphia: Lindsay & Blakiston. 1867.

This work comprises reports upon Physiology by Henry Power, Practical Medicine by Dr. Anstie, Surgery by Thomas Holmes, Ophthalmology by Thos. Windsor, Midwifery and the Diseases of Women and Children by Dr. Barnes, Medical Jurisprudence, Materia Medica and General Therapeutics and report on Public Health by Dr. C. Hilton Fagge.

These reports embody a great many interesting and instructive facts, and comprise the recent discoveries and advances made in all the various departments of medicine and surgery. The editors, who fully understand the real *status* of knowledge in their several departments have canvassed the ground, and we have thus presented the most interesting and important papers and views which are afforded by the progressive efforts of the profession in all countries. This work of about 500 pages comprises a vast amount of discussion and description, and should be in the hands of every member of the profession.

---

Spotted or Congestive Fever. By C. B. Coventry, M. D., Utica, N. Y.

This monograph is a careful digest of the existing knowledge of the symptoms, both general and special, pathology, diagnosis, prognosis and treatment of spotted fever, and constitutes a valuable addition to the scanty literature upon this obscure and often fatal epidemic.

Lawson on Injuries of the Eye.

This work comprises the minute and comprehensive description of all injuries of the organ of vision and of the best medical and surgical means of treatmeut. He considers the superficial injuries, the effects from scalds, burns and chemical agents, penetrating wounds and other injuries of the cornea and iris, traumatic cataract, capsular opacities and dislorations of the lens, foreign bodies within the eye, traumatic intraocular pressure and rupture of the globe, gun-shot injuries of the eye, sympathetic, ophthalmia, injuries of the orbit, and of the eyelids. It also contains abstract of the surgical reports of the Royal London Ophthalmic Hospital, Morefields, 1866, and Jaeger's Test-types. We have examined the work with great care, and believe it to be a valuable addition to our surgery of the eye. It is illustrated with numerous wood cuts, wherever these would add distinctness and value, and is published in a very neat and becoming manner.

---

Transactions of the Medical Society of the State of Kansas, for the year 1867.

Although the actual existence of this Society dates back but one year, we are pleased to observe the evidences of a vigorous life, exhibited in its transactions. The essays are able and instructive productions, the report of the Committee on Climatology by T. Sinks, M. D., and an essay on "The Malarial Miasm pervading Non-Malarial Diseases," by C. C. Sheyer, M. D., possessing high merit,

